# Optimizing Rehabilitation Outcomes for Stroke Survivors: The Impact of Speed and Slope Adjustments in Anti-Gravity Treadmill Training

**DOI:** 10.3390/medicina60040542

**Published:** 2024-03-27

**Authors:** Jung-Ho Lee, Eun-Ja Kim

**Affiliations:** Department of Physical Therapy, Kyungdong University, 815 Gyeonhwon-ro, Munmak-eup, Wonju-si 26495, Republic of Korea; leejh@kduniv.ac.kr

**Keywords:** stroke, speed, slope, rehabilitation, anti-gravity treadmill

## Abstract

*Background and Objectives:* This study explored the efficacy of customized anti-gravity treadmill (AGT) training, with adjustments in speed and incline, on rehabilitation outcomes for stroke patients, focusing on knee extensor muscle strength, joint angle, balance ability, and activities of daily living (ADLs). *Materials and Methods:* In this study, 30 individuals diagnosed with a stroke were divided into three groups. Experimental group 1 (EG1) underwent training without changes to speed and incline, experimental group 2 (EG2) received training with an increased incline, and experimental group 3 (EG3) underwent training with increased speed. Initially, all participants received AGT training under uniform conditions for two weeks. Subsequently, from the third to the sixth week, each group underwent their specified training intervention. Evaluations were conducted before the intervention and six weeks post-intervention using a manual muscle strength tester for knee strength, TETRAX for balance ability, Dartfish software for analyzing knee angle, and the Korean version of the Modified Barthel Index (K-MBI) for assessing activities of daily living. *Results:* Within-group comparisons revealed that AGT training led to enhancements in muscle strength, balance ability, joint angle, and ADLs across all participant groups. Between-group analyses indicated that EG2, which underwent increased incline training, demonstrated significant improvements in muscle strength and balance ability over EG1. EG3 not only showed significant enhancements in muscle strength, joint angle, and ADLs when compared to EG1 but also surpassed EG2 in terms of knee strength improvement. *Conclusions:* In conclusion, the application of customized AGT training positively impacts the rehabilitation of stroke patients, underscoring the importance of selecting a treatment method tailored to the specific needs of each patient.

## 1. Introduction

Stroke remains a leading cause of long-term disability worldwide, affecting millions of individuals each year [1]. It primarily occurs due to an interruption of blood flow to the brain, which can result from either an ischemic blockage or a hemorrhagic bleed. This disruption leads to neuronal death and, consequently, to a range of neurological impairments based on the affected brain region. Among the sequelae of stroke, physical disabilities, especially those affecting the lower extremities, are among the most common and debilitating. These impairments significantly impact an individual’s ability to perform daily activities, thereby affecting their independence and quality of life [2].

Stroke is a disease that causes long-term disabilities that reduce mobility and daily living abilities. In particular, the lack of motor ability in the lower extremities affects gait and balance maintenance, as well as increases the risk of falls. The decreased walking abilities of post-stroke hemiplegic patients are known to be a major factor in hindering an independent daily life. Moreover, the gait patterns of stroke patients are characterized by slow gait cycles and speeds, different stride lengths between the affected and unaffected sides, as well as short stance phases and relatively long swing phases on the affected side. Given that hemiplegic patients suffer from gait disturbances due to impairments in joint and muscle control that are necessary for normal gait, improving walking ability is considered the most important factor in rehabilitation [3].

In post-stroke rehabilitation, the timing of interventions is a key factor for stroke patients, and active treatment for stroke patients in the acute phase has a positive effect on their functional recovery. The functional level has the most recovery within three months of the onset of the disease and gradually improves between three to six months thereafter. In particular, active movement in the acute phase not only activates the brain and improves its potential but also helps improve the quality of life [4]. More specifically, clinically applied rehabilitation treatment methods to improve the gait of stroke patients include the following: joint stabilization exercises, muscle strengthening, and aerobic exercises; constraint-induced movement, mirror, and electrical therapies; task-oriented training; robot-assisted rehabilitation; and daily living activities. Various such treatment methods have been tried and proven effective [5,6,7].

Muscle strength, particularly in the lower extremities, is profoundly impacted by stroke, leading to significant functional impairments. The loss of strength in muscles responsible for supporting and moving the body can drastically affect a stroke survivor’s ability to perform basic movements, such as standing, walking, and maintaining balance [8]. This weakness is often more pronounced on one side of the body, corresponding to the brain hemisphere affected by the stroke. The asymmetry in muscle strength not only challenges the re-acquisition of walking skills but also increases the risk of falls and injuries [9]. Rehabilitation efforts aimed at enhancing muscle strength focus on targeted exercises that stimulate muscle activity and promote neural recovery, facilitating the gradual return of function and mobility [10]. Balance impairments are common among stroke survivors, stemming from a combination of muscular weakness, coordination deficits, and sensory losses. The ability to maintain balance is critical for walking and performing daily activities safely and independently. Post-stroke, individuals often experience difficulties in controlling their body’s position in space, especially when transitioning between movements or encountering uneven surfaces. Training programs that include balance exercises are essential in stroke rehabilitation, as they help to improve postural stability, reduce the risk of falls, and support the relearning of walking [11]. The gait of stroke survivors is typically characterized by alterations in joint angles, leading to deviations from normal walking patterns [12]. These alterations can include reduced knee flexion, improper hip rotation, and ankle dorsiflexion deficits, all of which contribute to an inefficient and often unsafe gait. The analysis of joint angles during walking provides critical insights into the biomechanical deficiencies that need to be addressed in rehabilitation [13].

Recently, various methods other than conventional treatment methods for gait improvement in stroke patients have been attempted, among which treadmill training is widely clinically used for gait function training. Treadmill training induces repetitive and regular steps by adjusting the angle and speed of the moving surface, enables dynamic motion during gait training, and provides a quick opportunity for gait activity to re-educate stroke patients regarding walking [14]. In addition, treadmill training not only provides a walking environment similar to level ground but also allows stroke patients to perform repetitive motions. Thus, this therapeutic intervention is known to be an effective method for improving neuroplasticity through motor control and learning. Further, treadmill training can safely improve patients’ walking ability more effectively than when they walk on level ground by enabling them to walk left and right symmetrically while supporting their body weight and reducing spasticity in their lower extremities [15,16].

Overall, treadmill training programs should be included in the clinical rehabilitation of stroke patients so that they can have an active and correct gait pattern. However, when applying such a program, it is necessary to adjust the optimal values of gait speed and stride length for each patient in order to recreate a spatiotemporal rhythmic gait pattern. Since the sensitivity of the central motor nerve pathway can be changed through the stimulation provided by treadmill training, its application as a method of rehabilitation could influence the improvement of balance ability and gait in stroke patients through the enhancement of proprioceptive input to the damaged nervous system [14,16]. 

The anti-gravity treadmill (AGT) represents an innovative approach within the realm of rehabilitation technologies, specifically designed to address lower extremity disabilities following a stroke. By leveraging adjustable body weight support, the AGT allows individuals to engage in walking and balance exercises with reduced weight bearing, thereby minimizing the risk of injury and facilitating an earlier start to mobility training [17]. The ability to customize the level of body weight support enables the AGT to accommodate the unique needs and rehabilitation progress of each patient, showcasing its versatility and effectiveness in the rehabilitation of stroke survivors. In addition to body weight support, the AGT’s capabilities extend to adjusting both the slope and speed of the treadmill, offering a comprehensive approach to gait training that can be tailored to challenge the patient appropriately. The effects of slope adjustments involve altering the gravitational forces acting on the body, which can help in strengthening specific muscle groups and improving postural control. This method is grounded in the principle that varying the walking surface incline can significantly influence the activation patterns of lower limb muscles, particularly the knee extensors and hip flexors, which are crucial for improving walking ability and stability post-stroke [18]. On the other hand, speed adjustments necessitate changes in the neuromuscular and cardiovascular responses, potentially enhancing endurance, gait speed, and overall functional mobility [19,20]. Furthermore, the variation in speed and slope introduces a dynamic aspect to the rehabilitation program, challenging patients’ balance and coordination in a controlled environment. This dynamic challenge is essential for retraining the neural pathways involved in gait and balance, enhancing proprioception and motor control, which are vital for preventing falls and improving walking confidence [21,22]. The purpose of this study is to delve into the impact of varying training protocols on the AGT in the rehabilitation of stroke patients, with a keen focus on the effects of speed increment training. To address the gap in understanding the comprehensive benefits of anti-gravity treadmill training with speed and slope adjustments for stroke survivors, this study hypothesizes that customized treadmill training, characterized by specific adjustments in speed and incline, will lead to significant improvements in muscle strength, balance, gait efficiency, and activities of daily living (ADLs) among stroke survivors. This exploration extends to understanding how adjustments in slope and speed, individually and in combination, can influence rehabilitation outcomes. Despite the AGT’s recognized benefits across various patient populations, the literature lacks comprehensive evidence on the optimal training protocols that maximize functional recovery post-stroke. Through this investigation, the study seeks to elucidate the distinct benefits of speed and slope increment training protocols, contributing valuable insights into the optimization of AGT-based rehabilitation strategies for enhancing the recovery and quality of life of stroke survivors.

## 2. Materials and Methods

### 2.1. Subjects

Thirty patients diagnosed with ischemic strokes based on magnetic resonance imaging by a rehabilitation medicine specialist were included in this study. Prior to the experiment, all subjects were given a detailed and comprehensive explanation about all the procedures, purposes, and procedures in the study, rights that they could exercise during the experiment, side effects that could result from the experiment, and compensation. Participants were recruited from an affiliated outpatient rehabilitation clinic and divided into three groups using a computer-generated sequence to ensure unbiased distribution. All procedures in this study were carried out in accordance with the Declaration of Helsinki after obtaining approval from the Institutional Review Board. All experimental procedures and protocols were approved by the research ethics committee following the guidelines of the university.

The inclusion criteria for the study subjects were patients with hemiplegia after being diagnosed with ischemic strokes; whose onset of stroke occurred less than six months ago; with an MMSE-K score of 24 or greater; and aged 50 to 80 years, regardless of gender. Meanwhile, the exclusion criteria for the study subjects were patients with severe spasms in the lower extremities; taking drugs or psychotropic drugs to relieve spasms; who have experienced a fall or fracture after a stroke; who refuse to receive treatment; to whom treadmill training cannot be applied; and with cardiorespiratory problems.

### 2.2. Design

The 30 subjects selected according to the inclusion/exclusion criteria were divided into three experimental groups as previously outlined. These groups included the anti-gravity treadmill training group (EG1 group), the group with increased treadmill slope (EG2 group), and the group with increased treadmill speed (EG3 group). Prior to their respective interventions, all groups received 30 min of proprioceptive neuromuscular facilitation to prepare for the training sessions ahead. After the initial two-week introduction to their specific training regimes, adjustments were made to the protocols for groups EG2 and EG3 while maintaining uniformity in exercise duration across all groups. The EG1 group continued with their treadmill training for an additional four weeks without any change to the incline or speed, with each session unified to last 30 min. The EG2 group’s treadmill training was modified to include an increased slope for the next four weeks, with each training session also set to last 30 min. Similarly, the EG3 group experienced an increase in treadmill speed, with their adjusted training sessions lasting 30 min at this enhanced speed for the subsequent four weeks.

These interventions were conducted three times a week for a total duration of six weeks, culminating in 18 sessions for each participant. To ensure the study’s progression and the participants’ safety, a physical therapist with over a decade of clinical experience was assigned to oversee and evaluate the therapeutic interventions. Before the start of each experimental session and measurement, subjects were thoroughly briefed on the usage of equipment, the methods of measurement, and the training program’s progression.

### 2.3. Intervention

For the EG1 group in this study, the intervention utilized an anti-gravity treadmill device (Alter-G, Fremont, QC, Canada). During the initial treadmill exercise session (2 weeks), minimal weight support was provided, ensuring the patients did not experience discomfort. In the subsequent training sessions (4 weeks), the weight support was adjusted weekly based on patients’ capabilities, under the therapist’s supervision. Additionally, patients were instructed to watch the monitor on the treadmill equipment for visual feedback. To maintain the safety of the training, the treadmill speed was initially set at 0.5 km/h. Each exercise session was conducted for a duration of 30 min, allowing for a focused and sustained period of rehabilitation activity tailored to patients’ progress and tolerance levels. For the EG2 group, the intervention involved the use of an AGT device, similar to Group 1. The primary distinction for Group 2 was the systematic increase in the treadmill’s incline to enhance the rehabilitation process. The initial session (2 weeks) began with minimal weight support, ensuring the patients’ comfort and without any incline adjustment, to familiarize the patient with the treadmill environment. As the sessions progressed (4 weeks), the incline was carefully adjusted on a weekly basis, in addition to the weight-bearing adjustments. The objective was to gradually introduce a gentle slope, starting from a 0% incline, with increments of 1% to 2% per week, depending on patients’ tolerance and capability. If patients experienced difficulty or discomfort, incline levels were reduced or maintained according to individual needs, with 5% being established as the final goal for the incline level to accommodate individual rehabilitation goals and physical capabilities. Sessions for Group 2 lasted 30 min each, providing ample time for acclimatization to the increased demands of incline walking under the constant supervision of a therapist. For the EG3 group′s intervention strategy also utilized the AGT. However, the focus for this group was on gradually increasing the walking speed, rather than adjusting weight support or incline. The intervention began (2 weeks) with minimal body weight support to ensure patient comfort and did not involve increasing the speed initially. In the subsequent sessions (4 weeks), starting from a basic speed of 0.5 km/h, the speed was gradually increased by 0.1 to 0.3 km/h each week, with a final goal of reaching 0.9 km/h to ensure progressive adaptation and improvement in walking speed for the rehabilitation process. The duration of each exercise session was set to 30 min.

Proprioceptive neuromuscular facilitation was performed in all groups before each intervention for the full 6-week duration of the study, and treatment patterns were applied for the lower extremities. For the proprioceptive neuromuscular facilitation of the lower limbs, the patient was instructed to take a supine position as comfortably as possible. After a stable posture was established, a pattern was applied in the following order: it started in the posture of hip joint extension–abduction–lateral rotation, knee joint extension, and ankle joint plantar flexion–inversion, followed by hip joint flexion–abduction–medial rotation, knee joint flexion, and ankle joint dorsal flexion–eversion, and ended with a return back to the starting position.

### 2.4. Outcome Measure

In this study, a pre-test and post-test were performed before and after the therapeutic interventions, respectively. All evaluations were made by a physical therapist who had more than 10 years of clinical experience. For the evaluation of the subjects, they were trained in advance to obtain the standards for measurement after undergoing regular education and attending meetings. In addition, to reduce errors in measurement, the therapist who performed the pre-evaluation for a patient also carried out the post-test.

#### 2.4.1. Muscle Strength Test of Knee Extensors

To perform a test to evaluate the muscle strength of affected legs, a manual muscle strength tester was used to measure each leg in kg units (Model01163, Lafayette, IN, USA). This tester can measure the range of 0 to 136.1 kg in increments of 0.2 kg at high intensity and the range of 0 to 22.6 kg in increments of 0.1 kg at low intensity, with a measurement error of ±1%. A chair with an adjustable sitting height was prepared so that the subject’s knee angle was 90° when sitting on the chair. With hips touching the edges of the chair, the subject pushed one leg forward at a time according to the evaluator’s instructions. Here, the subject was instructed not to lift their hips, and the test was started with both hands lightly placed on their knees. In this study, the pressure during the maximum isometric contraction of the knee extensors was measured. Subsequently, the knee extensors were measured after the subjects were instructed to straighten their knees with a pressure plate placed on the front of the ankle. Both legs were measured three times, and the average of these values was used as the measurement data.

#### 2.4.2. TETRAX Portable Multiple System

TETRAX (Sunlight Medical Ltd., Ramat Gan, Israel) equipment was used to test subjects’ static balance abilities. TETRAX is a diagnostic tool used to assess balance problems and fall risks. It measures balance and stability by recording fluctuations in vertical pressure using four independent and integrated force platforms. In addition, portable force plates are located on both sides of the subject to measure changes in weight placed on four points (two heels and two toes) to evaluate postural disturbance and thereby calculate postural variables. Since pressure changes are measured by installing independent force plates on the left and right toes and heels, this equipment can be used to evaluate balance ability and generate more information compared to that of general position recorders. 

In this study, the stability test index (STI) was determined using TETRAX. More specifically, STI is an indicator of the stability of the center of gravity, and it is defined by measuring changes in the pressure applied to each force plate. A lower STI value indicates a more stable postural balance, reflecting fewer fluctuations and greater control over body position. Conversely, an increased STI value suggests diminished stability, characterized by greater variability in the center of gravity and a reduced ability to maintain a steady posture.

#### 2.4.3. Gait Ability

For the kinesiology analysis of the three groups of stroke patients in this study, motion analysis was performed using the Dartfish program (Pro Suite, DfKorea, Hanam-si, Republic of Korea) before and 6 weeks after the treatment. To evaluate patients’ gait, they were instructed to walk on a flat path that was 10 m in length. A camera was installed 3 m away on the side. Before filming the video, black markers were attached to the greater trochanter of the femur, the lateral epicondyle of the knee, and the condyle of the ankle on the paralyzed side. Videos were recorded while the patient was walking, after which the medial angles of the knee joint at the time of heel off on the paralyzed side were measured during the gait cycle using Dartfish software. The choice was informed by the pivotal role the knee joint plays in the mechanics of gait, especially in the transition phases between swing and stance. During the gait cycle, the knee joint undergoes significant flexion and extension movements, which are crucial for maintaining gait efficiency, stability, and fluidity. In the evaluation of knee joint angles within our study, the measurement process specifically used exterior angles according to markers placed on participants. In this study, the averages of the three knee angles obtained through the analysis were used as the measurement data.

#### 2.4.4. Daily Living Activities

In this study, to evaluate the effects of factors, such as muscle strength, balance, and walking ability, on daily living activities, patients’ functional activities were evaluated using the Modified Barthel Index (MBI). As a tool to evaluate the functional level of basic daily living activities, the K-MBI was standardized after modifications and supplementations of some items to suit Korea. The MBI consists of a total of 10 items, and each item is scored on a 5-point scale with a total score of 100, in which 0 to 24 points indicate complete dependency, 25 to 50 points indicate maximum dependency, 50 to 74 points indicate partial dependency, 75 to 90 points indicate some dependency, and 91 to 99 points indicate independent activity.

### 2.5. Statistical Analysis

For the data of all variables obtained through this study, their means and standard deviations were calculated using the statistical program SPSS PC for Windows (version 18.0). Descriptive statistics were used to analyze the general characteristics of the subjects, and a one-way analysis of variance (ANOVA) was used to test homogeneity for subject characteristics between groups. A paired-sample *t*-test was conducted to analyze the differences between pre- and post-tests within groups, and a one-way ANOVA, analyzed using the average of the difference between the pre-test and the post-test, was performed to compare the treatment effects between the groups. Finally, a post hoc test was conducted using the LSD (least significant difference), with all statistical significance levels set at α < 0.05.

When the sample size calculation was conducted using the G*Power version 3.1 (http://www.gpower.hhu.de (accessed on 3 December 2022)) software, a G*Power analysis, with an α of 0.05 and a power of 80%, suggested a total of 30 participants would be adequate to identify significant differences among the experimental groups, given an estimated effect size of d = 0.8.

## 3. Results

Table 1 presents the general characteristics and pre-test homogeneity of the subjects across three experimental groups. There were no significant differences among the groups in terms of gender, age, height, weight, knee extensor strength, stability test index, knee joint angle, and K-MBI scores.

### 3.1. Knee Extensor Muscle Strengths on Paralyzed Sides

The data regarding the knee extensor muscle strengths of the lower extremities on the paralyzed side are presented below (Table 2). Across all three experimental groups, an improvement in the knee extensor muscle strength on the paralyzed side was noted post-experiment compared to the pre-experiment measurements (*p* < 0.05). Specifically, for EG1, the muscle strength increased from 14.40 kg before the experiment to 17.19 kg afterward. In EG2, the strength went from 15.35 kg to 16.24 kg. For EG3, an increase from 14.82 kg to 16.48 kg was observed. A one-way ANOVA was conducted to compare the degree of changes in muscle strength among the groups (*p* < 0.05). Post hoc analysis identified that EG2 and EG3 experienced more significant changes compared to EG1. Moreover, the changes in EG3 were found to be significantly greater than those in EG2 (*p* < 0.05).

### 3.2. TETRAX Portable Multiple System

The data on balance maintenance ability improvements across three experimental groups, both before and after interventions (Table 3), show significant progress in each group (*p* < 0.05). In EG1, which underwent treadmill training, the balance scores improved from an average of 23.93 points in the pre-test to 21.65 points in the post-test, indicating a significant change. EG2, which participated in increasing incline treadmill training, experienced an even more substantial improvement, with scores reducing from 24.72 to 21.79 points. EG3, engaging in increasing speed treadmill training, also showed improvement, with scores decreasing from 24.24 to 22.56 points. Analysis comparing the effects of these training methods on balance ability revealed significant differences between groups (*p* < 0.05), with post hoc analyses showing that EG2 improvements were noticeably greater than EG1 improvements.

### 3.3. Knee Joint Angle

The study compared the knee joint angles during heel off through gait analysis among the three experimental groups, both before and after the intervention, to observe changes (Table 4). In EG1, the knee joint angle decreased from 164.30 degrees in the pre-test to 161.60 degrees in the post-test, resulting in a change of 2.70 degrees, which was statistically significant (*p* < 0.05). EG2 saw a decrease from 163.30 degrees to 161.00 degrees, with a change of 2.30 degrees, which was also significant (*p* < 0.05). EG3 experienced a reduction from 165.30 degrees to 161.30 degrees, marking a change of 4.00 degrees, and this too was significant (*p* < 0.05). However, when comparing the magnitude of changes among the groups using a one-way ANOVA, the differences were not statistically significant (*p* = 0.56), indicating that while each group saw improvements, the extent of change did not significantly differ across the groups. Post hoc analysis suggested that experimental group 3 had a greater improvement than experimental group 1 despite the overall ANOVA result.

### 3.4. Daily Living Activities

In evaluating the impact of different interventions on subjects’ activities of daily living, an analysis of the total scores of the K-MBI was conducted (Table 5). Across all three experimental groups, increases in mean total K-MBI scores were observed post-experiment compared to pre-experiment (*p* < 0.05). In EG1, the K-MBI scores improved from an average of 39.60 in the pre-test to 42.00 in the post-test, showing a significant increase of 2.40 points. EG2, which participated in increasing incline treadmill training, experienced an improvement in scores from 42.90 to 46.30, a significant increase of 3.60 points, while EG3, undergoing increasing speed treadmill training, showed the most considerable improvement, with scores escalating from 39.90 to 44.70, an increase of 4.80 points. A one-way ANOVA was conducted to compare the improvement in ADLs across the groups, revealing a significant difference (*p* < 0.05), which indicated variability in the effectiveness of the interventions. Post hoc analysis identified that EG3 had a significantly greater improvement in ADLs compared to EG1, suggesting that increasing speed treadmill training was more effective in enhancing ADLs than the treadmill training alone.

## 4. Discussion

It plays a critical role in learning new things and forming memories and is an essential element in the functional restoration of stroke patients. More specifically, habituation in neuroplasticity is its simplest form and is a type of non-associative learning [23]. For example, when a weak pain stimulus is applied to a limb, there is an initial avoidance reflex, but when repeated stimulation is applied, there is a reflex action that no longer has an avoidance reflex. In addition, since the brain is active and complex, for neuroplasticity to occur, there must be repetitive practice accompanied by attention, and this is defined as user-dependent plasticity. Although neuroplasticity may occur in the natural neurological recovery process, its effect can be maximized only when there is continuous repetitive practice for motor learning and control [24]. The exploration of neuroplasticity’s pivotal role in the rehabilitation of stroke patients, as elucidated in our study, aligns with and extends the understanding of its fundamental importance in learning, memory formation, and functional restoration post-stroke. This study’s findings further highlight the necessity of repetitive, focused practice in rehabilitation to maximize neuroplasticity’s benefits for motor learning and control.

Recently, many studies have been conducted on the reorganization of cranial nerves in relation to treatment and learning after brain injuries. Previous studies have shown that extensive cortical reorganization occurs through neuroplasticity in people with central nervous system damage and that for use-dependent reorganization, the size of cortical representations in body parts increases proportionally to the amount of usage. By increasing the movement of body parts through intensive and repetitive training in an attempt to control their lack of use after a stroke, cortical reorganization is promoted [25]. As a result, the size of the cortical representation responsible for the function of movement is increased, which, in turn, can ensure the correct body movement. In addition, according to usage, cortical reorganization establishes the basis for the use and movement of a damaged body by converting normally learned unused body parts to those that can be used in daily life activities. Taken together, repetitive lower extremity movements based on the theory of brain neuroplasticity promote cortical reorganization in stroke patients, so they should be considered an important element when planning rehabilitation treatment programs and should not be omitted [26]. By adjusting the speed and incline of treadmill training, we observed significant improvements in knee extensor muscle strength, balance, joint angle, and activities of daily living, suggesting that these tailored exercises promote cortical reorganization and functional improvement in a manner that standard treadmill training does not. This supports the concept that intensive and repetitive training can increase the cortical representation of body parts used in therapy, leading to improved movement control and the re-acquisition of abilities essential for daily life.

To promote neuroplasticity in the rehabilitation process of stroke patients, harmonious and appropriate interactions between the sensory and motor systems are important [23]. However, in a significant number of stroke patients, proprioception of the lower limbs on the paralyzed side is reduced, and the spatial sensory feedback of the body does not function at the required level, resulting in increased disturbance of the body and reduced efficiency of movement. In addition, such proprioception impairments as experienced by stroke patients also influence factors essential for functional activities and cause various problems, including motor control impairment, gait disturbance, decrease in daily living activities, and increased risk of falls [4]. Of these, the gait disturbance pattern results in insufficient flexion of the knee joint during the swing and early stance phases, and stroke patients experience difficulties in shifting weight to the affected side, as well as in supporting weight during the stance phase. Our study’s results, demonstrating enhanced outcomes through customized AGT training, underscore the detrimental effects that reduced proprioception and spatial sensory feedback can have on a stroke survivor’s movement efficiency and balance. The tailored treadmill interventions likely provided enriched sensory feedback and forced repetitive, controlled movements, contributing to improved proprioception, reduced gait disturbances, and, consequently, decreased risk of falls. This aligns with existing research that identifies proprioceptive deficits as critical factors affecting functional activities and mobility in stroke patients [27].

Weakness in muscle strength after a stroke is closely associated with a decrease in functional performance. The weakness of the lower limb muscles, in particular, has a significant correlation with balance and walking abilities. Specifically, the weakened leg muscle strength on the paralyzed side of stroke patients causes asymmetry in the stance phase during walking and a decrease in gait ability due to a decrease in the gait speed, as well as in the length of the single-leg support phase [28]. In addition, muscles weakened due to paralysis not only have a reduced recruitment rate during muscle contraction but also have difficulty controlling contraction timing, resulting in the attenuation of simultaneous contraction. Since this attenuation of simultaneous contraction leads to a decrease in balance ability and increases the risk of falls, various muscle-strengthening exercises are currently being tested in clinical practice to achieve functional improvements in stroke patients [29].

Meanwhile, Folland et al. (2007) stated that the purposes of therapeutic muscle strengthening training are to recover from or prevent skeletal muscle defects and improve function, health, and the achievement of daily activities through neuromuscular adaptation responses [30]. In this context, Ouellette et al. (2004) reported that high-intensity resistance training increased muscle strength in stroke patients [31]. Further, Weiss, Suzuki, Bean, and Fielding (2000) observed a significant increase in muscle strength on both the affected and unaffected sides after stroke patients received progressive resistance training [32]. In addition, Morris et al. (2004)’s systematic review study showed that progressive resistance training for adult stroke patients is effective in reducing musculoskeletal disorders through muscle strengthening. Overall, these results suggest that muscle strength rehabilitation in stroke patients affects not only the recovery of muscle strength but also the ability to undertake daily living activities and gait function [33].

Among the various exercises applied in the rehabilitation of stroke patients, treadmill training is the most common intervention method in clinical practice. Specifically, it is used for gait rehabilitation, as it has the advantage of allowing stroke patients to perform appropriate aerobic exercises even in confined spaces. Additionally, treadmill training allows patients to perform task-oriented training repeatedly and can provide an environment similar to that of walking on level ground. For this reason, treadmill walking is widely practiced to improve the balance and gait of stroke patients [14,15,16]. In this context, previous studies found that gait training on treadmills elevates patients’ motivation for walking, increases their efforts to maintain constant gait speeds, and allows them to train gait patterns repeatedly. Other studies also reported that gait training on treadmills can selectively improve biomechanical gait factors in stroke patients and reduce energy consumption when they walk on the ground [34]. In addition, Ada et al. (2008) investigated the effects of gait training programs on a treadmill and on the ground on the gait ability of stroke patients. In the study, 26 subjects were selected, and training was conducted for four weeks. The results show that a statistically significant increase in walking speed and endurance was observed in the experimental group that received gait training on a treadmill [35]. 

Muscle weakness significantly impacts the functional ability of stroke patients, with a notable correlation between muscle strength and gait dynamics. The strength of the knee extensors and flexors, crucial for gait speed, also provides essential stability to the lower limbs during simultaneous contractions in the early stance phase [10]. This study reveals that EG2 and EG3, which underwent treadmill training with an increased incline and a gradual increase in speed respectively, experienced more substantial increases in knee extensor muscle strength compared to EG1. Such improvements in muscle strength, particularly observed in groups undergoing training with an increased incline and speed, can be attributed to neuromuscular adaptation and muscle hypertrophy. The heightened neuromuscular demands from the gradual increase in speed enhance motor unit recruitment and synchronization are critical for the efficient and powerful muscle contractions needed for knee extension. Moreover, the progressive overload from increased speed and incline serves as a catalyst for muscle hypertrophy, promoting muscle fiber growth and an increase in the cross-sectional area.

Adding to these findings, this study also explored changes in balance ability and knee joint angles. A notable outcome was that EG2 showed significant improvements in balance ability compared to EG1, with no significant difference between EG2 and EG3, indicating that both increased incline and speed contribute effectively to balance maintenance without any discernible difference between the two methods. Furthermore, a significant difference in knee joint angle changes was observed between EG3 and EG1, underscoring the impact of treadmill training with a gradual increase in speed on enhancing joint mobility and stability. The authors believe that the observed increases in knee extensor muscle strength, alongside improvements in balance ability and modifications in knee joint angles, underscore the combined effects of neuromuscular adaptation and muscle hypertrophy. These physiological adaptations, driven by the mechanical stress and metabolic demands of tailored treadmill training, not only promote muscle strength but also enhance overall mobility and stability in stroke patients. This approach, emphasizing both muscle strength and functional abilities, such as balance and joint mobility, validates the effectiveness of customized treadmill training in the rehabilitation process for stroke patients, highlighting its role in addressing the multifaceted challenges posed by stroke-induced impairments.

The profound impact of lower extremity disabilities on the daily living activities of stroke patients cannot be overstated. Muscle weakness and reduced mobility, common sequelae of stroke, significantly impair a patient’s ability to perform essential tasks, such as walking, climbing stairs, and transitioning from sitting to standing [36]. These challenges are primarily due to compromised knee extensor strength and flexibility, which are pivotal for maintaining lower extremity strength, optimizing joint angles, and ensuring balance. The resultant instability during exercise and movement not only hinders the efficiency of gait but also elevates the risk of falls, further exacerbating the vulnerability of stroke survivors to injuries and diminishing their independence [8]. The disability patterns of stroke patients are remarkably diverse and intricately related. More specifically, it is difficult to control and treat the numerous disabilities that occur after a stroke using only a fragmentary therapeutic approach [1,2]. 

In this study, a comprehensive approach employing multifaceted methods, including the adjustment of speed and incline, was undertaken to assess the effectiveness of zero-gravity treadmill training in enhancing the activities of daily living (ADLs) among stroke patients. The intervention was characterized by its utilization of an AGT, designed to reduce the load on the lower extremities, thereby facilitating a more accessible and safer training environment for individuals recovering from stroke. The research findings indicate that all participant groups experienced improvements in their ADL capabilities following the intervention, underscoring the efficacy of AGT training in promoting functional recovery. Notably, the data revealed a nuanced outcome in the comparative analysis between the experimental groups. EG3, which underwent training with an increased speed, demonstrated statistically significant improvements in ADLs.

Drawing on the findings of our study, it becomes evident that tailored treadmill training, emphasizing gradual increases in incline and speed, can markedly enhance strength and balance. The resultant improvements in muscle strength are crucial for fostering a more stable and efficient gait, thereby diminishing the likelihood of falls and promoting greater independence in movement. We posit that the training’s beneficial impacts on balance and knee joint angles are instrumental in facilitating safer and more assured mobility during daily routines. This enhanced mobility extends beyond mere ambulation, empowering stroke survivors to more confidently and safely engage in a broad spectrum of activities within their homes and communities.

However, limitations of this study include a relatively small sample size of 30 stroke survivors, which may limit the generalizability of the findings to the broader population of stroke survivors. Additionally, the intervention and evaluation took place over a short period of 6 weeks and did not capture the long-term impact of the AGT training protocol. In addition, some patients complained of dizziness during exercise. Future research in the field of stroke rehabilitation utilizing AGT training should adopt a more comprehensive approach by integrating a variety of training parameters, such as resistance levels and employing different types of treadmills, to thoroughly evaluate their impact on rehabilitation outcomes. Additionally, longitudinal studies with extended follow-up periods should be conducted to gain deeper insight into the ongoing effectiveness and sustainability of the benefits derived from AGT training.

## 5. Conclusions

This study aimed to assess the efficacy of customized treadmill training interventions, including modifications in speed and incline, on the rehabilitation outcomes of stroke patients, specifically focusing on the enhancement of knee extensor muscle strength, balance, joint angle, and activities of daily living. Utilizing the innovative approach of AGT training, the study explored the potential benefits of reducing lower extremity load during rehabilitation exercises, thereby facilitating safer and more effective training sessions for individuals recovering from stroke. The findings of this research unequivocally demonstrate that all experimental groups experienced significant improvements in their ADL capabilities, knee extensor muscle strength, and balance following the interventions. These enhancements are critical for the recovery process, as they directly impact a patient’s ability to perform everyday tasks, contribute to their independence, and improve their quality of life. Notably, the study revealed that the groups subjected to AGT training with an increased incline and speed (EG2 and EG3) showed more pronounced improvements in knee extensor muscle strength compared to the group that received standard treadmill training (EG1). In addition, EG2 showed significant improvement in balance ability, and EG3 showed significant improvement in knee angle and ADLs compared to EG1. In conclusion, this study contributes valuable insights into the optimization of stroke rehabilitation practices, emphasizing the significance of customized, progressive treadmill training in improving muscle strength, balance, joint angle, and ADLs.

## Figures and Tables

**Table 1:** General characteristics and pre-test homogeneity of the subjects.

	EG1	EG2	EG3	*p*
Gender (female/male)	4/6	3/7	4/6	0.866
Age (year)	63.00 ± 4.52	65.10 ± 7.37	63.70 ± 5.43	0.723
Height (cm)	163.10 ± 8.01	162.80 ± 6.84	164.80 ± 8.21	0.824
Weight (kg)	58.805 ± 6.39	59.30 ± 11.17	59.70 ± 8.66	0.975
Knee extensor strength (kg)	15.25 ± 1.27	15.00 ± 1.68	14.56 ± 1.05	0.528
Stability test index (point)	23.93 ± 2.61	24.72 ± 2.43	24.24 ± 3.16	0.813
Knee joint angle (°)	164.30 ± 4.92	163.30 ± 4.87	165.30 ± 5.69	0.692
K-MBI (score)	39.60 ± 5.44	42.90 ± 6.34	39.90 ± 3.81	0.322

EG1: experimental group 1 (treadmill training), EG2: experimental group 2 (increasing incline treadmill training), EG3: experimental group 3 (increasing speed treadmill training), mean ± SD: mean ± standard deviation.

**Table 2:** The comparison of strengthening of knee extensor muscle in the affected side.

	Pre-Test	Post-Test	Changes (Pre–Post-Test)	*t*	*p*
EG1	15.25 ± 1.27	16.18 ± 1.67	−0.93 ± 0.59	−4.916	0.001 *
EG2	15.00 ± 1.68	16.60 ± 1.24	−1.60 ± 0.75	−6.669	0.000 *
EG3	14.56 ± 1.05	17.48 ± 1.76	−2.92 ± 1.01	−9.095	0.000 *
*F*			15.660		
*p*			0.000 **		
post hoc			EG3, EG2 > EG1 EG3 > EG2		

EG1: experimental group 1 (treadmill training), EG2: experimental group 2 (increasing incline treadmill training), EG3: experimental group 3 (increasing speed treadmill training), mean ± SD: mean ± standard deviation, * *p* < 0.05 (between pre-test and post-test), ** *p* < 0.05 (among groups), unit: kg.

**Table 3:** The comparison of balance maintenance ability.

	Pre-Test	Post-Test	Changes (Pre–Post-Test)	*t*	*p*
EG1	23.93 ± 2.61	21.65 ± 2.91	2.28 ± 1.28	5.631	0.000 *
EG2	24.72 ± 2.43	21.79 ± 2.49	2.93 ± 0.92	10.030	0.000 *
EG3	24.24 ± 3.16	22.56 ± 3.01	1.68 ± 0.68	7.707	0.000 *
*F*			3.950		
*p*			0.031 **		
post hoc			EG2 > EG1		

EG1: experimental group 1 (treadmill training), EG2: experimental group 2 (increasing incline treadmill training), EG3: experimental group 3 (increasing speed treadmill training), mean ± SD: mean ± standard deviation, * *p* < 0.05 (between pre-test and post-test), ** *p* < 0.05 (among groups), unit: point.

**Table 4:** Comparison between groups of knee joint angle during heel off through gait analysis.

	Pre-Test	Post-Test	Changes (Pre–Post-Test)	*t*	*p*
EG1	164.30 ± 4.92	161.60 ± 6.00	2.70 ± 1.82	4.669	0.001 *
EG2	163.30 ± 4.87	161.00 ± 4.64	2.30 ± 0.94	7.667	0.000 *
EG3	165.30 ± 5.69	161.30 ± 6.07	4.00 ± 1.76	7.171	0.000 *
*F*			3.222		
*p*			0.56		
post hoc			EG3 > EG1		

EG1: experimental group 1 (treadmill training), EG2: experimental group 2 (increasing incline treadmill training), EG3: experimental group 3 (increasing speed treadmill training), mean ± SD: mean ± standard deviation, * *p* < 0.05 (between pre-test and post-test), unit: angle.

**Table 5:** Comparison of activities of daily living between groups through the total score of K-MBI.

	Pre-Test	Post-Test	Changes (Pre–Post-Test)	*t*	*p*
EG1	39.60 ± 5.44	42.00 ± 4.49	−2.40 ± 1.83	−4.129	0.003 *
EG2	42.90 ± 6.34	46.30 ± 5.07	−3.60 ± 1.57	−6.815	0.000 *
EG3	39.90 ± 3.81	44.70 ± 2.58	−4.80 ± 1.93	−7.856	0.000 *
*F*			4.542		
*p*			0.020 **		
post hoc			EG3 > EG1		

EG1: experimental group 1 (treadmill training), EG2: experimental group 2 (increasing incline treadmill training), EG3: experimental group 3 (increasing speed treadmill training), mean ± SD: mean ± standard deviation, * *p* < 0.05 (between pre-test and post-test), ** *p* < 0.05 (among groups), unit: score.

## Data Availability

The datasets generated during the current study are available from the corresponding author upon reasonable request.
